# Gallstone Ileus Diagnosed During Endoscopic Evaluation: A Case Report

**DOI:** 10.7759/cureus.113003

**Published:** 2026-07-20

**Authors:** Soukaina Essadiqi, Fatima Zahra Belabbes, Imane Boutaleb Joutei, Khalid Sair, Imane Ben El Barhdadi

**Affiliations:** 1 Gastroenterology, Mohammed VI University of Sciences and Health (UM6SS), Mohammed VI Faculty of Medicine, Casablanca, MAR; 2 Gastroenterology, Mohammed VI Foundation for Health Sciences (FM6SS), Casablanca, MAR; 3 General Surgery, Mohammed VI University of Sciences and Health (UM6SS), Mohammed VI Faculty of Medicine, Casablanca, MAR; 4 General Surgery, Mohammed VI Foundation for Health Sciences (FM6SS), Casablanca, MAR

**Keywords:** biliary lithiasis, bilio-digestive fistula, gallstone small bowel obstruction, gastroenterology and endoscopy, urgent laparotomy

## Abstract

Gallstone ileus is a rare complication of calculous cholecystitis, resulting from the migration of a gallstone through a bilio-digestive fistula into the gastrointestinal tract, where it may cause mechanical obstruction. Diagnosis is often delayed due to a nonspecific clinical presentation.

We report the case of a 73-year-old man with a history of unoperated vesicular lithiasis who presented with intense epigastric pain, intractable vomiting, and clinical and biological signs of dehydration. After intravenous rehydration, esophagogastroduodenoscopy revealed a fistulous orifice in the duodenal bulb with a gallstone emerging through it (an uncommon and directly informative diagnostic finding) suggestive of a cholecystoduodenal fistula. A non-contrast CT scan confirmed a jejunal obstruction. The patient underwent laparotomy with stone extraction, duodenal suture closure, and cholecystectomy, with a favorable outcome.

This case highlights the value of upper gastrointestinal endoscopy as a complementary diagnostic tool in gallstone ileus, particularly when the stone remains accessible in the proximal gastrointestinal tract, and underscores the need for a high index of suspicion in elderly patients with atypical obstructive symptoms.

## Introduction

Biliary ileus or gallstone ileus is a rare entity responsible for intestinal obstruction. It results from the migration of a gallstone through a bilio-digestive fistula, formed as a consequence of chronic cholecystitis-related inflammation eroding the adjacent bowel wall [1,2], followed by its impaction within the gastrointestinal tract. It is most often a complication of calculous cholecystitis and accounts for 1%-4% of the causes of intestinal obstruction [1]. Morbidity is high in elderly and frail patients.

The clinical presentation is atypical, leading to diagnostic delays and delayed management. The diagnosis is established by abdominal CT scan, which demonstrates the classic triad of pneumobilia, intestinal obstruction, and ectopic localization of a gallstone. Treatment is surgical and consists of enterotomy with stone extraction, resection of the bilio-digestive fistula, with or without cholecystectomy [2].

Diagnostic delay is common, as initial clinical and even radiological findings may be nonspecific, and standard ultrasonography frequently fails to identify the fistula or the ectopic calculus. Upper gastrointestinal endoscopy is rarely used in this context and is not part of the standard diagnostic algorithm, which makes its contribution to establishing the diagnosis in our patient particularly noteworthy. 

We report a case of biliary ileus in a 73-year-old patient admitted with epigastric pain and intractable vomiting, in whom upper gastrointestinal endoscopy made it possible to establish the diagnosis.

This article was previously presented as an abstract at the European Society of Gastrointestinal Endoscopy (ESGE) Days 2025 on April 3, 2025.

## Case presentation

We report the case of a 73-year-old man whose only medical history was known gallstone disease that had not been surgically treated. He presented to the emergency department with severe epigastric pain associated with intractable vomiting of undigested food material (non-bilious), without associated hematemesis.

On clinical examination, the patient was tachycardic at 96 beats per minute, normotensive (134/66 mmHg), eupneic with an oxygen saturation of 95% on room air, and afebrile, with signs of dehydration evidenced by decreased skin turgor. The patient presented with clinical signs suggestive of acute upper gastrointestinal obstruction, including intractable vomiting and a fasting succussion splash on abdominal examination, without complete cessation of bowel movements, consistent with a partial obstructive syndrome.

Abdominal examination revealed a slightly distended abdomen that was soft on palpation, with a fasting succussion splash; the hernial orifices were free. Laboratory investigations showed acute renal failure with a creatinine level of 18.6 mg/L and hypokalemia at 2.4 mmol/L. The remainder of the workup revealed leukocytosis at 15,250/mm³ with neutrophil predominance and an elevated C-reactive protein (CRP) level of 45 mg/L (Table 1). The acute renal failure and hypokalemia were interpreted as consequences of prolonged vomiting and dehydration, prompting immediate intravenous rehydration and electrolyte correction before further investigation. The leukocytosis and elevated CRP, in the absence of fever, raised suspicion of an underlying intra-abdominal inflammatory or obstructive process rather than a purely infectious one, guiding the decision to pursue upper endoscopy despite an unremarkable abdominal ultrasound.

**Table 1:** Laboratory findings at admission.

Biological parameter	Value	Reference range
Creatinine	18.6 mg/L	7–13 mg/L
Urea	1.37 g/L	0.15–0.45 g/L
Serum sodium	135 mmol/L	135–145 mmol/L
Serum potassium	2.4 mmol/L	3.5–5.4 mmol/L
Lipase	34 IU/L	8–78 IU/L
Hemoglobin	17.2 g/dL	12.5–16.5 g/dL
White blood cell count	15,250/mm³	3,800–10,000/mm³
Neutrophils	12,570/mm³	1,600–5,900/mm³
Platelets	302,000/mm³	140,000–385,000/mm³
C-reactive protein (CRP)	45 mg/L	< 8 mg/L

**Figure 1:**
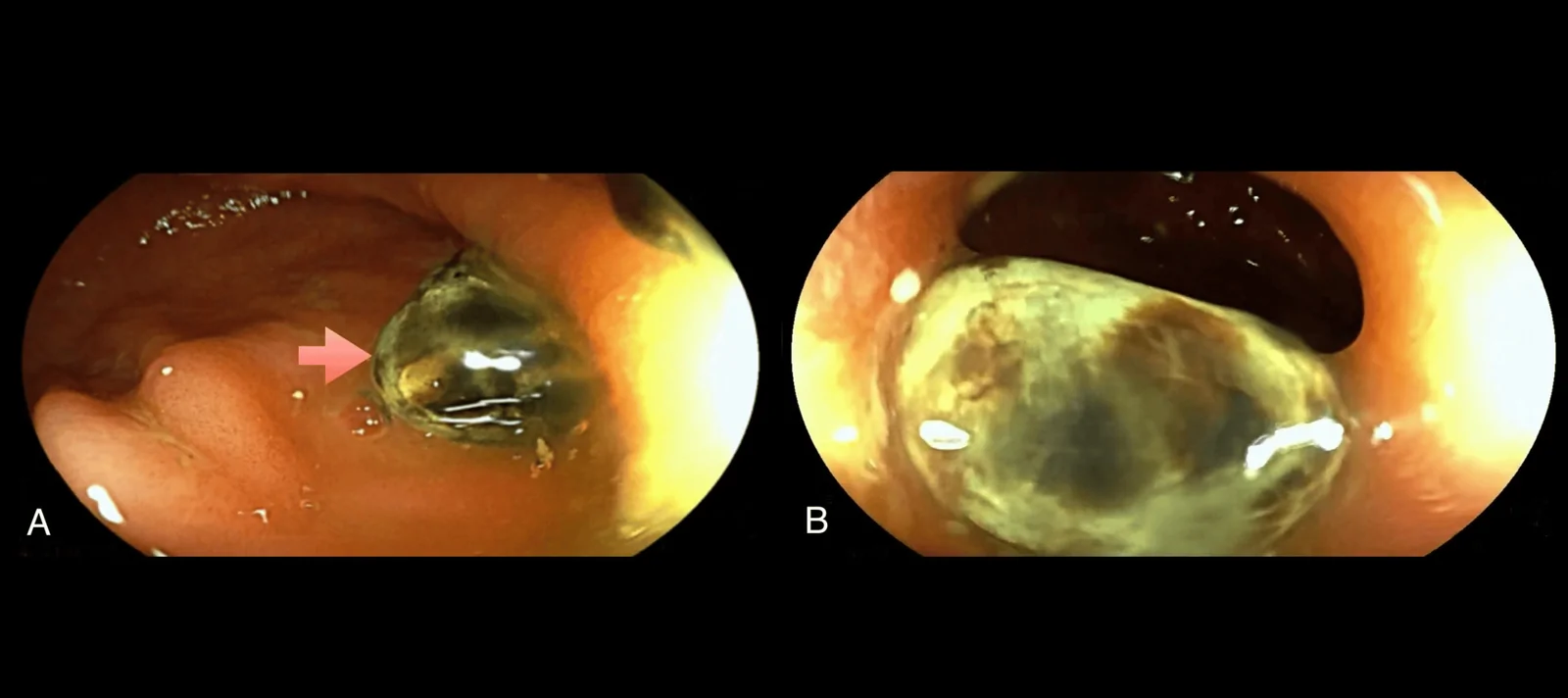
A and B : Endoscopic images showing a gallstone emerging from the duodenal bulb (red arrow).

After initial stabilization, an esophagogastroduodenoscopy was performed and revealed gastric stasis with purulent and fetid contents, as well as visualization of a fistulous orifice at the level of the posterior duodenal bulb (D1, first portion of the duodenum), with passage of a gallstone through it, endoscopically suggestive of a bilio-digestive fistula (Figure 1).

**Figure 2:**
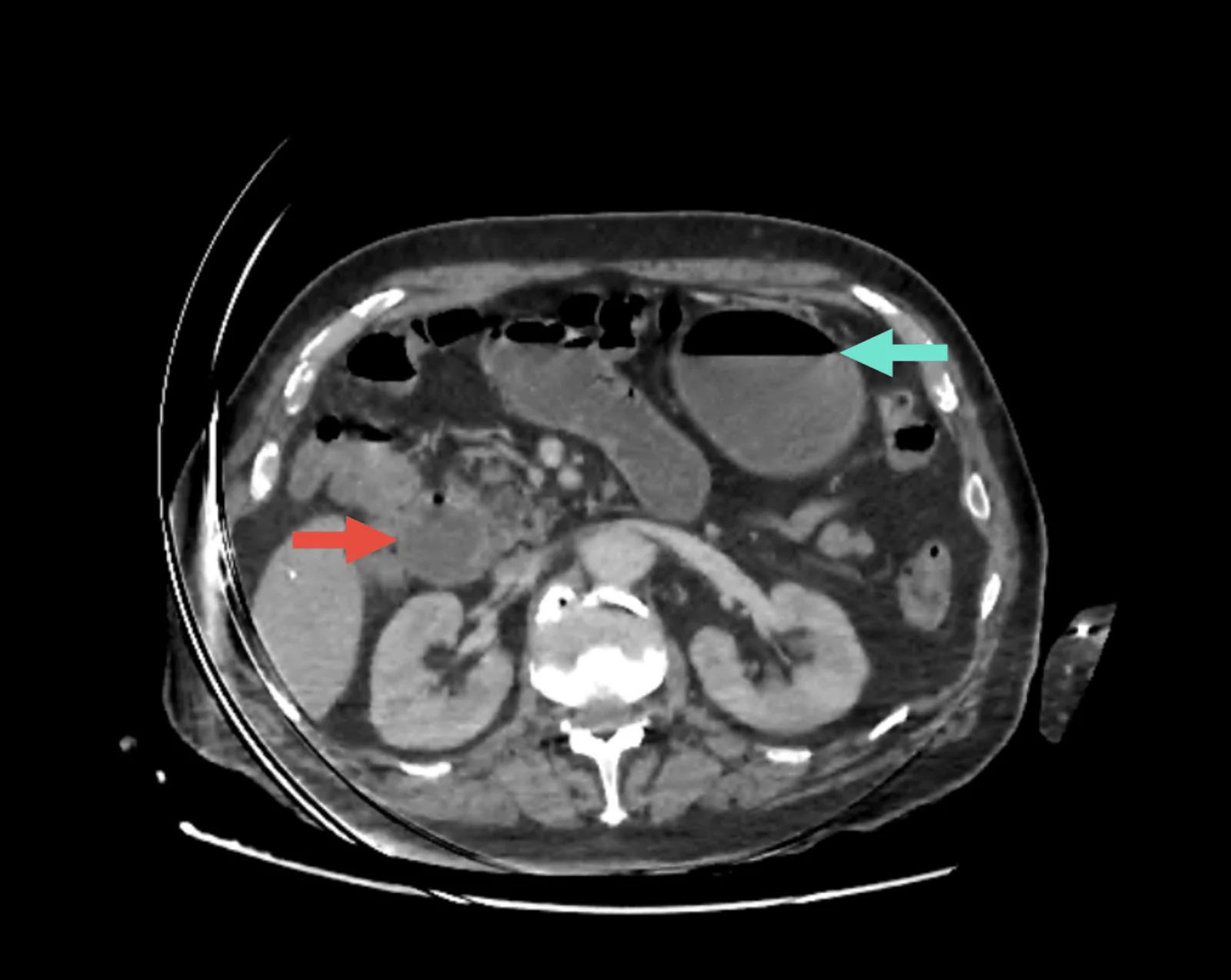
Axial non-contrast CT scan showing an intraluminal gallstone located in the jejunum associated with an air–fluid level. Red arrow: intraluminal gallstone; Blue arrow: air–fluid level

A non-contrast abdominopelvic CT scan demonstrated gastroduodenal distension upstream of an intraluminal proximal jejunal obstruction, with moderate dilatation of the left intrahepatic bile ducts (Figure 2). The combination of proximal jejunal obstruction with intrahepatic bile duct dilatation on CT, together with the endoscopic finding of a fistulous orifice and a stone in the duodenal bulb, was considered sufficient to establish the diagnosis of gallstone ileus with cholecystoduodenal fistula without further imaging, and prompted urgent surgical referral.

**Figure 3:**
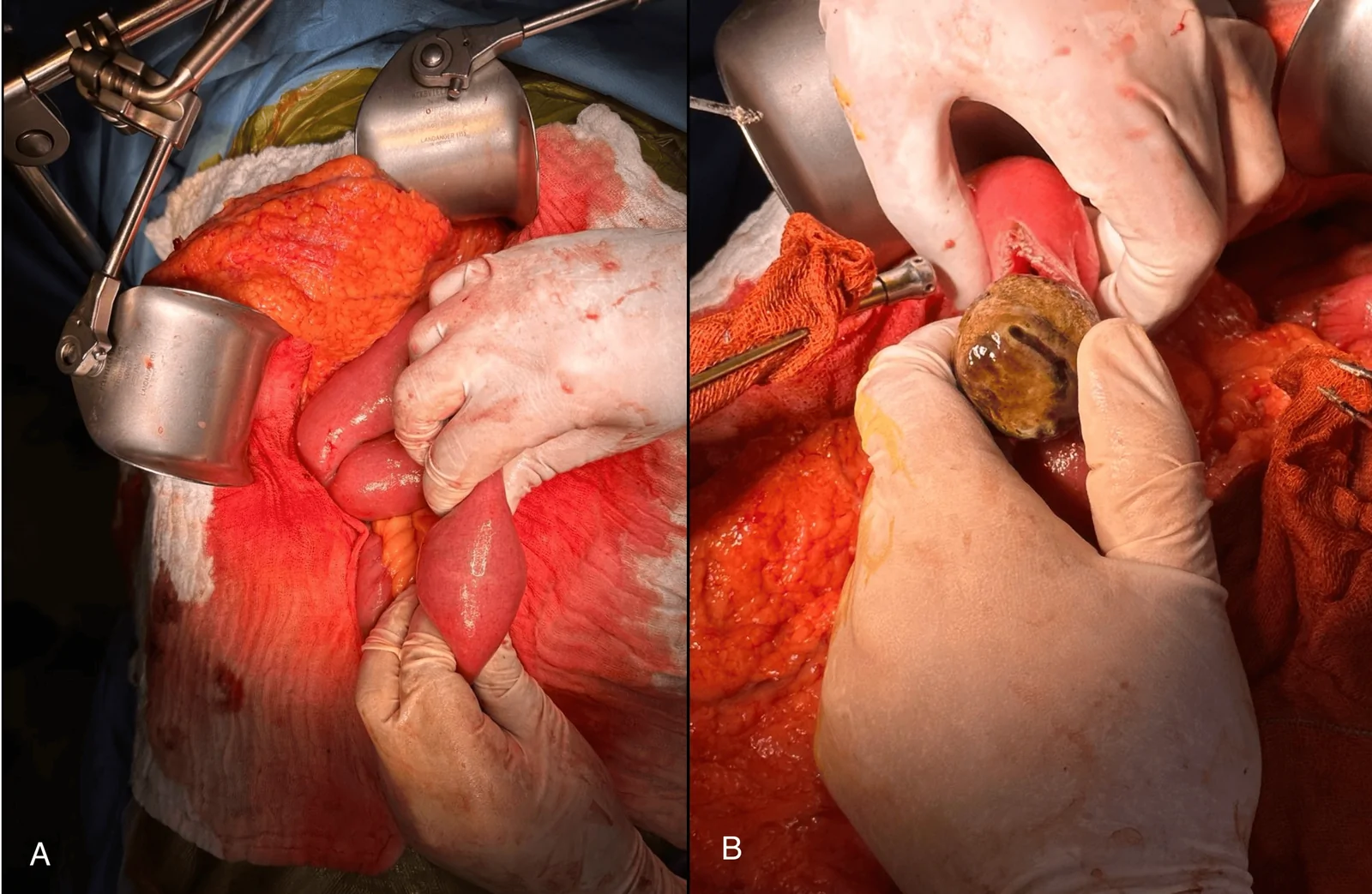
Intraoperative view of the gallstone, impacted in the duodenum, extracted by enterotomy.

**Figure 4:**
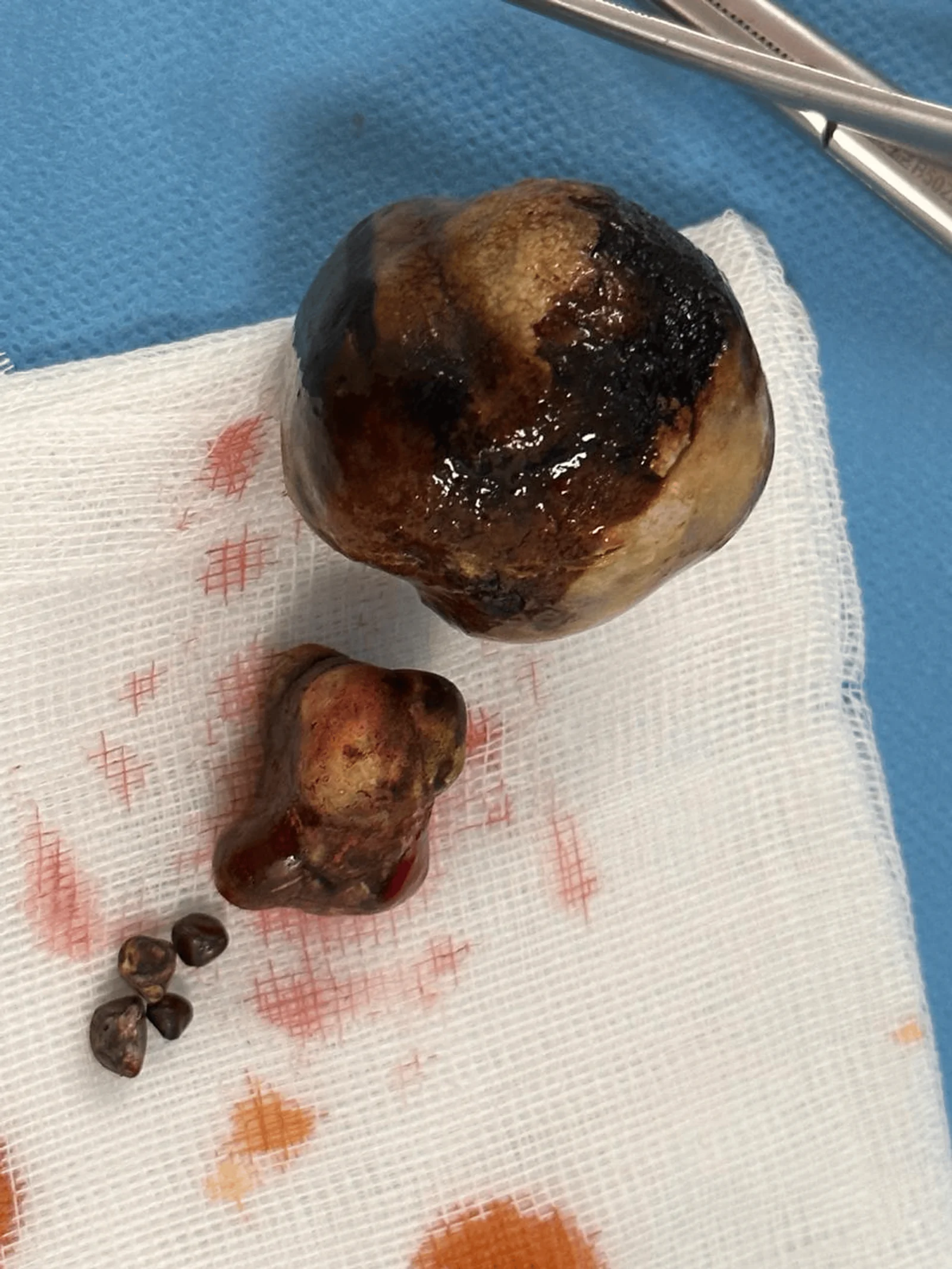
A giant gallstone and micro-calculi removed by enterotomy.

The patient was taken to the operating room for laparotomy. A large gallstone measuring 4 cm and associated micro-calculi located in the jejunum were found, along with a cholecystoduodenal fistula. The patient underwent enterotomy with stone extraction (Figures 3, 4), gastrojejunostomy resection and anastomosis, closure of the duodenal defect, and cholecystectomy.

Postoperative recovery was uneventful, with a favorable outcome, gradual resumption of oral intake, and discharge on postoperative day 7.

## Discussion

Biliary ileus is a rare condition resulting from ileal obstruction caused by a gallstone originating from a cholecystointestinal fistula. It accounts for 1%-4% of the causes of intestinal obstruction and shows a female predominance [1].

The pathophysiology is based on repeated episodes of cholecystitis, which increase pericholecystic inflammation, causing the gallbladder wall to adhere to an adjacent hollow viscus (most commonly the duodenum), and the ensuing chronic inflammation and local ischemia progressively erode both walls, creating a cholecystoenteric fistula through which a gallstone can migrate into the bowel lumen [1,2]. In 10%-20% of cases, the ectopic gallstone becomes impacted within the gastrointestinal tract, resulting in an obstructive syndrome [2].

Biliary ileus occurs in 0.3%-0.5% of calculus cholecystitis but may also occur in the absence of a bilio-digestive fistula following endoscopic sphincterotomy, either immediately or several months later [3]. Rare cases of biliary ileus have been reported in patients who have previously undergone cholecystectomy [3]. Morbidity reaches 12%-18% in elderly and comorbid patients [1].

Clinically, the presentation is atypical and variable, consisting of an unusual obstructive syndrome associated with vomiting, abdominal pain, and cessation of bowel movements. However, diagnosis is often delayed due to diagnostic wandering and may sometimes be made intraoperatively [4, 5].

Abdominal CT scan allows diagnosis in 93% of cases, demonstrating Rigler’s triad in 20%: pneumobilia, intestinal obstruction, and ectopic localization of a gallstone [6]. CT imaging also helps rule out the presence of additional stones and identify the location of the fistula, which is most commonly cholecystoduodenal. These fistulas result from prolonged inflammation, particularly in chronic or recurrent cholecystitis [4]. The fistulous orifice may occasionally be visualized endoscopically, contributing to the diagnosis [7].

In our patient, the diagnosis was initially established by upper gastrointestinal endoscopy through visualization of the gallstone and the fistulous orifice. Unlike the majority of reported cases, in which diagnosis relies primarily on CT imaging and the fistula is inferred rather than directly visualized [3, 5], our case is notable for the direct endoscopic identification of both the fistulous orifice and the migrating gallstone prior to its impaction. This diagnostic pathway, though uncommon, illustrates a potential complementary role for upper endoscopy in atypical presentations of gallstone ileus, particularly when the stone remains accessible in the proximal gastrointestinal tract before descending further.

The location of the gallstone depends on its size and the diameter of the digestive lumen. Stones measuring between 2 and 10 cm are likely to cause obstruction, with an average size of 4.3 cm [3]. They are most frequently located at the ileocecal junction but may also be found in the jejunum, ileum, duodenum (Bouveret’s syndrome), or colon [4]. In our patient, the stone was located in the duodenum, at the posterior wall of the bulb.

Management is primarily surgical by laparotomy. It is an emergency and should be performed early, although conservative management may be considered in elderly patients with spontaneous symptom improvement [8]. Two surgical approaches are possible: enterotomy alone with stone extraction or enterotomy combined with repair of the bilio-digestive fistula, followed by cholecystectomy in one or two stages [9]. In inoperable patients, endoscopic extraction after stone fragmentation by lithotripsy may be attempted [10].

Our patient underwent laparotomy with enterotomy and stone extraction, closure of the duodenal defect, gastrojejunostomy, and cholecystectomy, with an uneventful postoperative course and favorable outcome.

## Conclusions

Biliary ileus remains an uncommon but serious cause of intestinal obstruction, associated with delayed diagnosis and substantial morbidity and mortality. Clinical manifestations may be nonspecific, underscoring the importance of maintaining a high index of suspicion, particularly in elderly or frail patients presenting with obstructive symptoms. Although rare, it should be systematically considered in the differential diagnosis of bowel obstruction. Prompt surgical management remains the cornerstone of treatment and is essential to improving patient outcomes.
